# Gas-chromatography and UV-spectroscopy of Hymenoptera venoms obtained by trivial centrifugation

**DOI:** 10.1016/j.dib.2018.03.101

**Published:** 2018-03-27

**Authors:** Eduardo G.P. Fox, Meng Xu, Lei Wang, Li Chen, Yong-Yue Lu

**Affiliations:** a Red Imported Fire Ant Research Center, South China Agricultural University, Guangzhou 510642, PR China; b Chinese Academy of Sciences, Institute of Zoology, State Key Laboratory of Integrated Management of Pest Insects and Rodents, Beijing 100101, PR China

## Abstract

This paper summarises gas-chromatography (GC–MS) and preliminary UV-spectroscopy analyses data of fresh, unmodified venom of aculeate hymenopterans (ants, bees, wasps), mainly focusing on red imported fire ants. No solvents nor fractionation were used at any point, which is a novel approach to describing integral toxins cocktails as proposed by Fox et al. (2018a) [1] 10.1016/j.toxicon.2018.02.050 where these results are discussed in deeper details. Herein we focus on further characterising the obtained venom extracted through a novel approach. Pertaining raw data is accessible from Fox et al. (2018b) [2] 10.17632/cpnscw2gkc.1 including further relevant information regarding the used insects, machinery settings, chemical standards.

## Highlights

• A method for milking crude venom from arthropods by trivial centrifugation is described.
• The new method enabled discovery of new molecules in the venom of fire ants.
• Pilot tests with other species illustrate immediate potential for novel insights into venom composition.
• Due to its simplicity the method can be easily adapted to other arthropods.

## Graphical abstract

**Figure:**
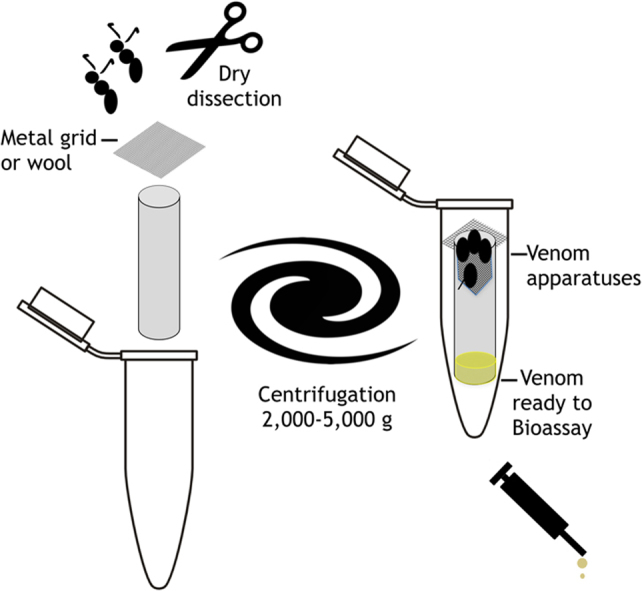

**Specifications Table**

**Table:** 

Subject area	*Immunology and Microbiology, Chemistry, Biology, Entomology, Toxinology*
More specific subject area	*Animal Toxins of Medical Importance, Bioassays of Natural Products, Immunotherapy*
Type of data	*Table, image (GC–MS chromatogram)*
How data was acquired	*Pure venoms were obtained by centrifugation using a simple adaptation described in cited references. The venoms were injected directly into Agilent gas chromatographs according with settings described in references. The resulting chromatogram files were analysed using the software OPENCHROM v.1.0.*
Data format	*Raw chromatogram files in various formats*
Experimental factors	*Tweezers, centrifuge tubes, a centrifuge are necessary.*
Experimental features	*Insects were collected from the university campus, identified using specific taxonomic characters, anesthesized with either CO2 or ethyl-acetate, and crudely dissected for venom-containing body parts. These tissues were transferred while fresh and alive into an adapted glass insert in centrifuge tubes. Mild centrifugation promptly yielded pure, milked venom off the collected insects. The obtained venoms were injected directly without solvents.*
Data source location	Campus of South China Agricultural University, Wushan Road, Tianhe, Guangzhou, People's Republic of China. Insects obtained from various locations inside the campus.
Data accessibility	All pertaining raw files are deposited in a public database available at http://dx.doi.org/10.17632/cpnscw2gkc.1

**Value of the data**

• The enclosed chromatograms of natural-state venoms are novel, and such data is rarely published as raw chromatograms are seldom made available by researchers in this research field.
• A micro-volume spectrophotometre is employed for the first time for the chemical analysis of venomous secretions.
• Raw chromatograms available from the referenced database [2] relate with future publications, and the described methods are open for critical evaluation and revisitation by peers interested in the chemistry of natural products.
• The methods proposed can facilitate the identification of novel compounds of animal origin.

## 1 Data

**Fig. 1:**
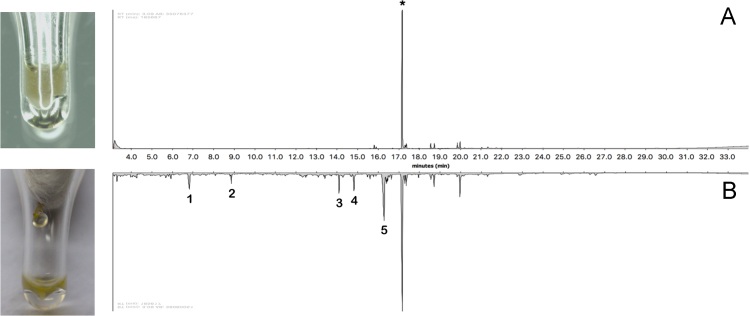
GC-MS chromatograms of pure honeybee venoms collected by centrifugation. A. The venom of Apis cerana; B. the venom of A. mellifera. Asterisk marks the main shared compound, eicosenol. Peaks on lower chromatogram are possibly species-specific pheromones, listed on Table 1. Raw spectra available at [2].

**Fig. 2:**
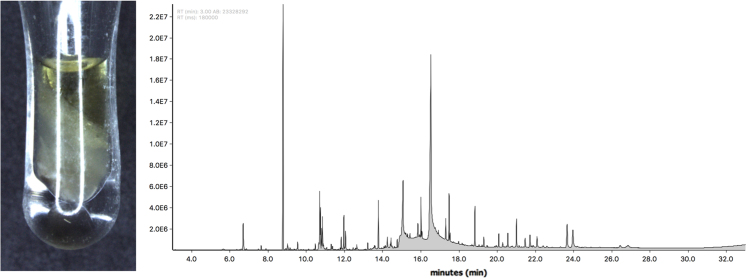
GC-MS chromatogram for pure Asian wasp Polistes jokahamae venom collected by centrifugation. Only the upper oily phase of the extract was analysed as the lower phase was too dense to inject and likely not volatile. The obtained result is a complex mixture of esters, fatty acids, hydrocarbons (not shown herein). Raw chromatogram files available at [2].

**Fig. 3:**
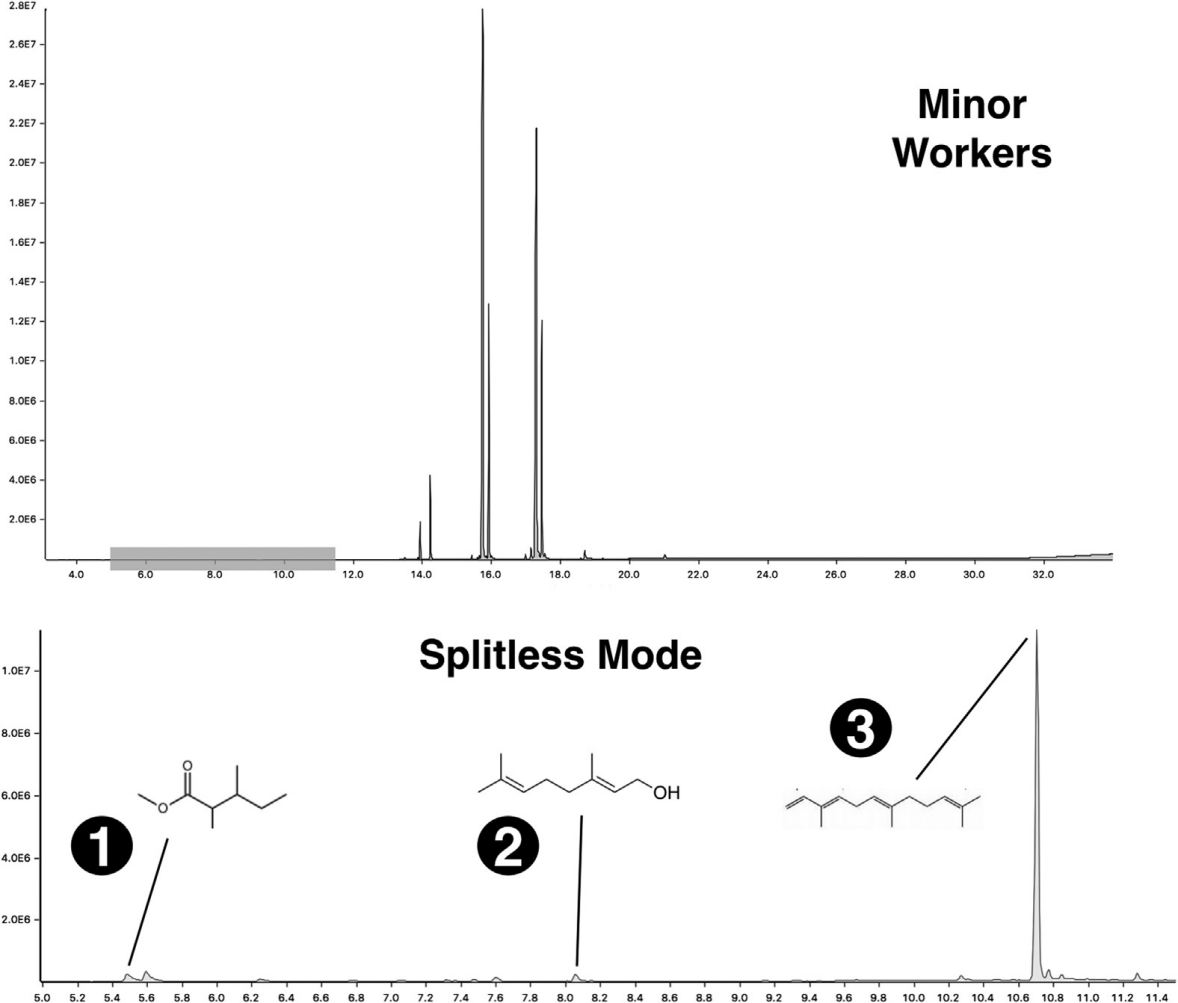
Fire ant venom collected by centrifugation, as viewed by GC-MS chromatograms. Upper panel: Crude venom chromatograms of S. invicta minor workers acquired in 1:100 split ratio. Lower panel: Magnified chromatogram from the shaded time interval in the upper panel, as obtained by splitless mode injection. Peaks are minor compounds included in pure venom, where some were tentatively identified as: (1) L-isoleucine methyl ester; (2) geraniol; (3) a-farnesene. For details on the spectra of all compounds find the original chromatogram files in [2].

**Fig. 4:**
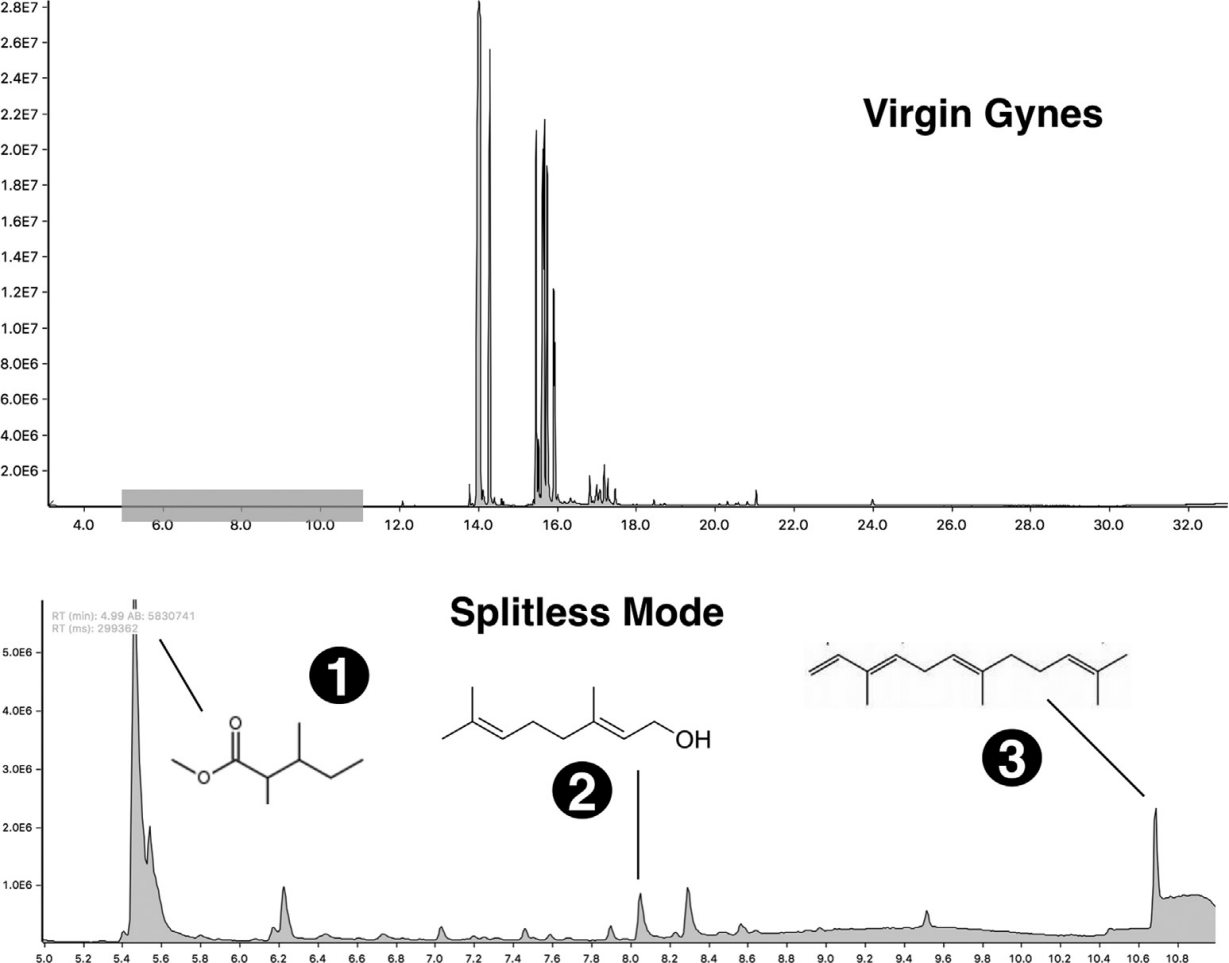
Fire ant queen venom collected by centrifugation, as viewed by GC-MS chromatograms. Upper panel: Crude venom chromatograms of S.invicta virgin gynes acquired in 1:100 split ratio. Lower panel: Magnified chromatogram from the shaded time interval in the upper panel, as obtained by splitless mode injection. Peaks are minor compounds included in pure venom, where some were tentatively identified as: (1) L-isoleucine methyl ester; (2) geraniol; (3) a-farnesene. For details on the spectra of all compounds find the original chromatogram files in [2].

**Fig. 5:**
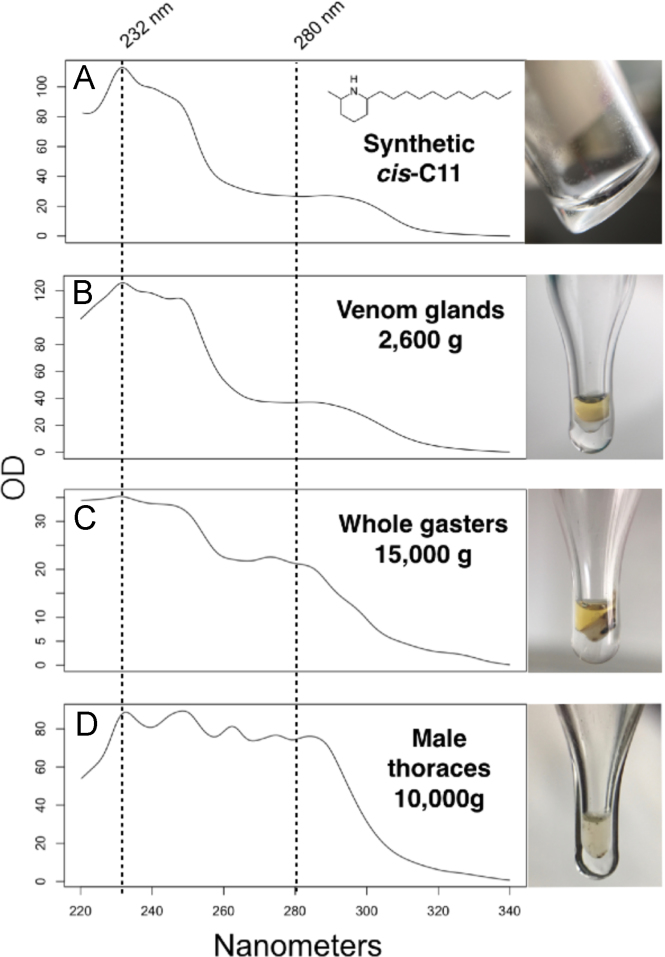
Spectrophotometric UV analysis of synthetic and natural fire ant extracts. A relative purity check for alkaloids was attempted following the same principle used with DNA and protein extracts, as described in [3], assuming maximum absorbance of solenopsin alkaloids at OD232 and of proteins at OD280. A - Synthetic isosolenopsin A, OD232:OD280 = 4.24; B - Venom obtained by gentle centrifugation of venom glands apparatuses of S. invicta minor workers, OD232:OD280 = 3.42; C - Whole-gaster extract obtained by high centrifugation of S. invicta minor workers, OD232:OD280 = 1.66; D - Thoracic hemolymph obtained by high centrifugation of S. invicta virgin males, OD232:OD280 = 1.19.)

The presented results are general analyses of GC–MS chromatograms obtained from crude venoms without any chemical or physical manipulation (e.g. chromatography, solubilisation). Such information is still rare in the scientific literature of venom toxins, as authors usually obtain modified venom fractions from dissecting animals and/or soaking venom-containing tissues in solvents. Herein venom collected by centrifugation of two species of honeybees (Fig. 1) , one social wasp (Fig. 2) , and three castes of red imported fire ants are presented (Fig. 3, Fig. 4, Fig. 5) . All pertaining raw files are publicly available at 10.17632/cpnscw2gkc.1.

**Table 1:** Tentative identification of differential peaks from Fig. 1 found in pure venoms of Apis cerana.

	R.T.*	Height	Area	Library search top hit result	Annotation
PEAK 1	6.824	5362825	210127283	4H-Pyran-4-one, 2,3-dihydro-3,5-dihydroxy-6-methyl-	Honey antimicrobial
PEAK 2	8.862	3457185	81305776	5-Fluoro-2-methylaniline	ID uncertain
PEAK 3	14.091	6559636	114746695	Z-5-Nonadecene	Ant trail pheromone
PEAK 4	14.822	5777014	168493006	n-Hexadecanoic acid	Pheromone
PEAK 5	16.409	3209663	57883330	Octadecanoic acid	Wax component

The venoms of fire ants are analysed and discussed in further details at [1].

## 2 Experimental design, materials and methods

Aggressive aculeate insects used were obtained around the university campus of South China Agricultural University. They were anesthesized with either CO2 or ethyl acetate (details at [1]). Isolated living venom-containing body parts were amassed at the numbers of 3–10 inside an adapted basket made of either glass wool or fine metal mesh in a glass vial insert. The glass insert was inserted into a trivial centrifuge tube and allocated into a centrifuge Eppendorf 5417R set to 28 °C and centrifuged at short 30–60 s cycles to 2000–6000 g. The same was done with mutilated alitrunks from fire ant males.

You will see that liquids collect at the bottom of the glass insert during centrifugation. Gently relocate the tissues inside the basket in order to change their orientation as to push out more liquids (e.g. venom or hemolymph) through the stopper mesh. Centrifuge again. After ca. 8 cycles the amount of collected liquid at the bottom of the glass insert stabilised (collected amounts presented in [1]).

The obtained venoms and hemolymph were injected directly without dilution to Agilent GC–MS systems according with the method described in [1] and available directly from [2].

Furthermore, venom of fire ants, synthetic solenopsin alkaloids, and hemolymph were submitted to spectrophotometric analysis using a Nano-300 AllSheng micro-spectrophotometer. The equipment was blanked empty, and about 1.0 μl of liquid extract was applied to the reading pedestal, and scanned > 3 times to ensure readings are stable. Pedestals were cleaned with acetone and distilled water between each use. The same solvents were also used as controls to test for reading stability and consistency between samples.
