# Supplementary material for: Gas-chromatography and UV-spectroscopy of Hymenoptera venoms obtained by trivial centrifugation
Source: Data Brief. 2018 Mar 27;18:992–8. doi: 10.1016/j.dib.2018.03.101 (PMC5996826; doi:10.1016/j.dib.2018.03.101)

Ethical Statement for Toxicon

I testify on behalf of all co-authors that our article submitted to Toxicon:

Title: Speedy milking of fresh venom from Aculeate hymenopterans

All authors: Fox, Eduardo GP; Meng, Xu; Wang, Lei; Chen, Li; Lu, YongYue

1) this material has not been published in whole or in part elsewhere;
2) the manuscript is not currently being considered for publication in another journal;
3) all authors have been personally and actively involved in substantive work leading to the manuscript, and will hold themselves jointly and individually responsible for its content.

Date:
2018-01-06

Corresponding author's signature:

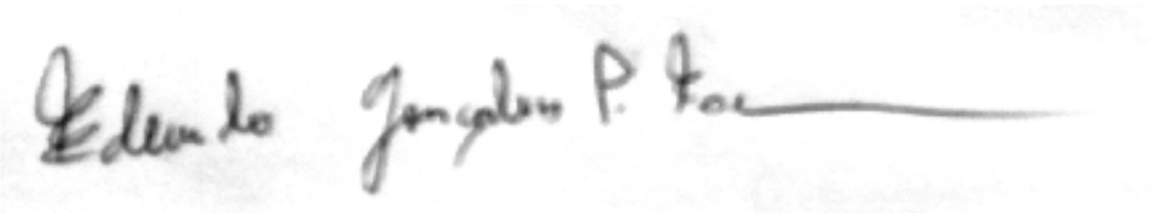

Supplement: Supplementary file 2 — Supplementary material [file mmc2.pdf]
